# Genomic surveillance of *Plasmodium falciparum* and *Plasmodium vivax* cases at the University Hospital in Tegucigalpa, Honduras

**DOI:** 10.1038/s41598-020-78103-w

**Published:** 2020-12-01

**Authors:** Hugo O. Valdivia, Fredy E. Villena, Stephen E. Lizewski, Jorge Garcia, Jackeline Alger, Danett K. Bishop

**Affiliations:** 1 Department of Parasitology, U.S. Naval Medical Research Unit 6 (NAMRU-6), Lima, Peru; 2 NGO PRISMA, Lima, Peru; 3 Hospital Escuela, Tegucigalpa, Honduras

**Keywords:** Malaria, Parasitology

## Abstract

Malaria continues to be an important health problem in Honduras despite major progress achieved reducing its incidence in the last two decades. In a context of case reduction, continuing surveillance of parasite diversity and drug resistance is an important component to assist effective malaria control strategies and support risk assessments. In this study, we employed next generation sequencing on collected *Plasmodium vivax* and *P. falciparum* samples from the Hospital Escuela (University Hospital) in Honduras between 2005 and 2017. Hospital Escuela is the main public health hospital in Honduras and receives suspected malaria cases from endemic regions within the country. The resulting sequencing data was used to assess complexity of infections, parasite population structure, parasite diversity and drug resistance profiling. All *P. vivax* samples and all autochtonous *P. falciparum* samples were monoclonal and presented a low intra population diversity (π = 0.25 and 0.07, respectively). Genotyping of drug resistance markers showed that three *P. falciparum* samples presented the chloroquine resistant haplotype SVMNT on *pfcrtr* (positions 72–76). Epidemiological data suggested that two of these samples were imported cases from Africa whereas the third one was a local case. Three suspected imported cases (two of which were also *pfcrt* mutants) presented the pfmdr1 86Y mutation that further enhances the CQ resistant genotype. No evidence was found for kelch13 artemisinin resistance associated mutations nor parasite genetic background mutations. Discriminant analysis of principal components and phylogenetic analysis showed two *P. vivax* and two *P. falciparum* parasite sub-populations with limited recombination between them. It also confirmed the closer relationship of the three imported cases with African strains. Our findings showed that local Honduras *P. falciparum* strains do not hold CQ resistance polymorphisms which aligns with clinical data reported by the country and supports the continuity of CQ based treatment in Honduras. In addition, our findings highlight the need of using genomic approaches to provide key information about parasite biology including drug resistance, population structure and HRP2/HRP3 deletions which are becoming relevant as the country move towards elimination.

## Introduction

Malaria remains as a major public health threat throughout tropical endemic regions causing more than 219 million cases in 2017^1^. Although malaria incidence has recently increased in South America, most countries in the Central America region have experienced a decrease of more than 20% since 2016^1^. This progress has fostered efforts towards malaria elimination in Central America and the island of Hispaniola by 2030^2^. In Honduras, malaria prevalence has consistently dropped in more than 86% since 2010 resulting in less than 2000 cases reported in 2017^1^. Out of those, *Plasmodium vivax* and *P. falciparum* are responsible of 90% and 10% of all malaria cases reported, respectively^1^. Treatment for uncomplicated infections still relies on the use of a combined therapy of chloroquine in addition to primaquine that is used for *P. falciparum* gametocytes and *P. vivax* hypnozoites. These drugs are still active despite more than 60 years of use and the widespread prevalence of chloroquine (CQ) resistance reported in most endemic regions of South America^3^.

It is known that genomic plasticity and diversity of *Plasmodium* constitute a barrier for malaria elimination programs^4^ since they can result in the rapid development of resistance against antimalarial drugs^4^. Furthermore, human migration and commercial activities can provide means for the introduction of drug resistance or virulent genotypes into areas with no indigenous resistant parasites^5–7^.

Therefore, monitoring changes in parasite genotypes can help identify early the emergence of resistant parasites and thereby informing the local health authorities to implement appropriate responses to prevent spread of resistant strains. This is especially important in the context of Honduras given previous reports of CQ resistance in neighboring countries such as Nicaragua^8^ and the lack of recent studies on genetic diversity and drug resistance^9^.

Furthermore, genomic studies can provide useful information for malaria elimination which is especially important for Honduras such as assess changes in circulating parasite populations and transmission dynamics, explore the prevalence of HRP2/HRP3 deletions and study molecular markers that can be used to determine the introduction or re-introduction of malaria parasites as the country move towards elimination^10^.

In this study, we performed a comparative analysis of 16 *P. falciparum* and 23 *P. vivax* samples collected in the University Hospital (Hospital La Escuela, HE) in Tegucigalpa Honduras between 2005 and 2017. Genomic data was used to explore the population structure of *P. falciparum* and *P. vivax* and identification of drug resistance genotypes.

## Materials and methods

### Sample collection

The study used de-identified specimens and data collected at the University Hospital in Tegucigalpa, Honduras between 2005 and 2017. The HE is the main public health hospital in Honduras and received suspected malaria cases from multiple regions within the country. As part of regular laboratory activities, blood spots were collected on a 3 mm Whatmann filter paper from each malaria microscopy confirmed patient at the time of diagnosis along with clinical and basic epidemiological data.

The protocol for this study (NAMRU6.2018.0002) was reviewed and approved by the Research Administration Program of the Naval Medical Research Unit-6 (NAMRU-6). Informed consent was not required for this secondary research since it does not meet the definition of research involving human subjects per US Code of Federal Regulations, 32 CFR Part 219-PROTECTION OF HUMAN SUBJECTS, section 219.104 Exempt research. Additionally, all methods were carried out in accordance with relevant guidelines, regulations and good laboratory practices.

### DNA extraction and LAMP PCR

DNA from each filter paper was extracted using the DNeasy Blood & Tissue kit (Qiagen) according to the manufacturer’s protocol. The resulting parasite DNA was screened for malaria by a Malachite green LAMP PCR (MG-LAMP) using a previously described method with slight modifications^11,12^. Briefly, MG-LAMP was performed in a 20 µL reaction volume that contained 5 µL of template DNA in 2× in-house reaction buffer (40 mM Tris–HCl pH8.8, 20 mM KCl, 16 mM MgSO_4_ , 20 mM (NH_4_)_2_SO_4_ , 0.2% Tween-20, 1.6 M Betaine, 2 mM of dNTP’s each), 0.25 µL of 1:400 SYTO 9 dye, 8 units of Bst Polymerase (New England Biolabs, Ipswich, MA) and 0.004% Malachite Green dye (MG). The genus specific LAMP assay targeted the *Plasmodium* mitochondria, the *P. falciparum* specific LAMP assay targeted the *Plasmodium* 18S subunit ribosomal RNA (ssrRNA) gene whereas the *P. vivax* specific assay targeted the Pvr64 gene (Table 1).

**Table 1:** Primers for genus and species-specific LAMP assays.

Target	*Plasmodium *genus	*P. falciparum*	*P. vivax*
	Mitochondria	18sr DNA gene	R64 gene
References	Polley et al.^13^	Yamamura et al.^14^	Patel et al.^15^
FIP	GGT GGA ACA CAT TGT TTC ATT TGA TCT CAT TCC AAT GGA ACC TTG	CAC CTA GTC GGT ATA GTT TAT GGT GCC TAA TCT ATT TCC ATT AAT	ATA TGG TCT CTC GAC ACG GCC AAA TTG CCA TCA TCT TCA C
BIP	GTT TGC TTC TAA CAT TCC ACT TGC CCG TTT TGA CCG GTC ATT	GTA GCA TTT CTT AGG GAA TGT TGG CCC CAG AAC CCA AAG ACT TTG A	TGT GCC CAC CCA CAT ACT TGG GGA AAT GTT AAT GGG GAT GT
F3	TCG CTT CTA ACG GTG AAC	GAG GTG AAA TTC TAA GAT TTT CT	TCT GTT GGT GGA GTA GAT CC
B3	AAT TGA TAG TAT GAG CTA TCC ATA G	TTC CGT CAA TTC TTT TAA CTT TC	CCT ACG TTT TGG TGA ATC G
LPF	CAC TAT ACC TTA CCA ATC TAT TTG AAC TTG	GGT ATC TGA TCG TCT TCA CTC CC	AGG CTA CTT CTT TTG CTC C
LPB	TGG ACG TAA CCT CCA GGC	GAA TTG CTT CCT TCA GTA CCT TA	ACT TAC AGT GCT GTA GAG A

The amplification reaction was performed at 63 °C for 60 min using a mini heat block (Gene Mate, Bio Express, Utah, US) and results were visually inspected by two independent readers after 15 min post amplification. All samples were processed first by the genus-screening LAMP assay and then by the species-specific assays for *P. falciparum* and *P. vivax*.

### Sequencing and SNP genotyping

Genotypes for drug resistance loci, genetic barcodes, and parasite speciation were derived from multiplexed amplicon sequencing performed at the Wellcome Sanger Institute. In brief, extracted DNA was selectively whole genome amplified (sWGA)^16,17^ and multiplex PCRs were performed on the amplified DNA. For. *P. falciparum,* two multiplexes of 69 and 68 amplicons targeted drug resistance loci and genetic barcodes while a third multiplex contained two amplicons used for parasites speciation (https://www.malariagen.net/resource/29). For *P. vivax*, a multiplex PCR with 114 targets was performed on the amplified DNA. A second round of PCR was done on both *P. falciparum* and *P. vivax* multiplexes which incorporated Illumina flow-cell adapters and sample indexing barcodes. Products were size selected by SPRI-beads and then multiplexes were pooled before sequencing on an Illumina MiSeq. Reads were binned by sample index and genotypes were called per sample by aligning to a *P. falciparum* 3d7 or *P. vivax* Sal-1 amplicon reference. For *P. falciparum*, barcodes were created by concatenated genotypes at 101 SNPs spread across the genome. For *P. vivax*, the resulting reads were used to create sample barcodes derived from a previously published list of loci^18^. SNPs within the barcodes were represented by their respective nucleotides (A,T,C and G), as missing (X) or as heterozygous (N).

### Data analysis and complexity of infections

Barcoding data was cleaned using the “poppr” R package prior to genetic analysis in order to secure that only high quality data remains^19^. The parameters used comprised excluding samples with greater than 20% missing SNPs and excluding positions with greater than 20% missing calls.

Complexity of infections were assessed using the COIL and Real McCOIL tools in each sample^20,21^. These methods use the proportion of heterozygous calls in order to estimate COI under a Markov Chain Monte Carlo (MCMC) framework. COI is represented as the estimated number of individual parasites within a single infection and serves to classify them as monogenomic or polygenomic.

### Population diversity

The population barcode diversity (π) was calculated as previously described^18^. Briefly, barcode diversity was calculated as the mean of differences between each pair of samples across the population divided by the total number of assayed SNPs. The variability of barcode π values was assessed with 10,000 iterations of nonparametric bootstrapping. This method allows to estimate population diversity values ranging from low to high differentiation (0 to 1).

### Discriminant analysis of principal components and phylogenetic analysis

In order to provide an additional estimation of the parasite subpopulation, the resulting barcode was used for discriminant analysis of principal components using the R adegenet package^22^. Based on data from the cases that suggested that some of these samples were imported from Africa, we conducted a phylogenetic analysis. For this purpose, we employed jModelTest 2.1.5^23^ to carry out statistical selection of the best-fit models according to the Bayesian information criterion. Phylogenetic reconstruction was carried out using a maximum likelihood approach implemented in PhyML v3.0^24^ with 1000 bootstrap under the TPM2 substitution model selected by jModelTest 2.1.5^23^. Two African *P. falciparum* strains were used to root the tree in order to explore the relatedness of the “imported” and local cases. The phylogenetic tree was visualized in Figtree (http://tree.bio.ed.ac.uk/software/figtree/).

Additionally, a phylogenetic network was constructed including 25 sequences from Colombia^25^, 20 from one of our study sites in Peru and 20 from Sudan^26^ in order to assess the relationship among haplotypes of the Honduras samples. These analyses were carried out in PopART^27^ using the median-joining algorithm^28^.

### Disclaimer

The views expressed in this article are those of the author and do not necessarily reflect the official policy or position of the Department of the Navy, Department of Defense, nor the U.S. Government. Some authors of this manuscript are military service members and employees of the U.S. Government. This work was prepared as part of their official duties. Title 17 U.S.C. §105 provides that “Copyright protection under this Title is not available for any work of the United States Government”. Title 17 U.S.C. §101 defines a U.S. Government work as a work prepared by a military service member or employee of the U.S. Government as part of that person’s official duties.

## Results

### Sample collection and data cleaning

**Figure 1:**
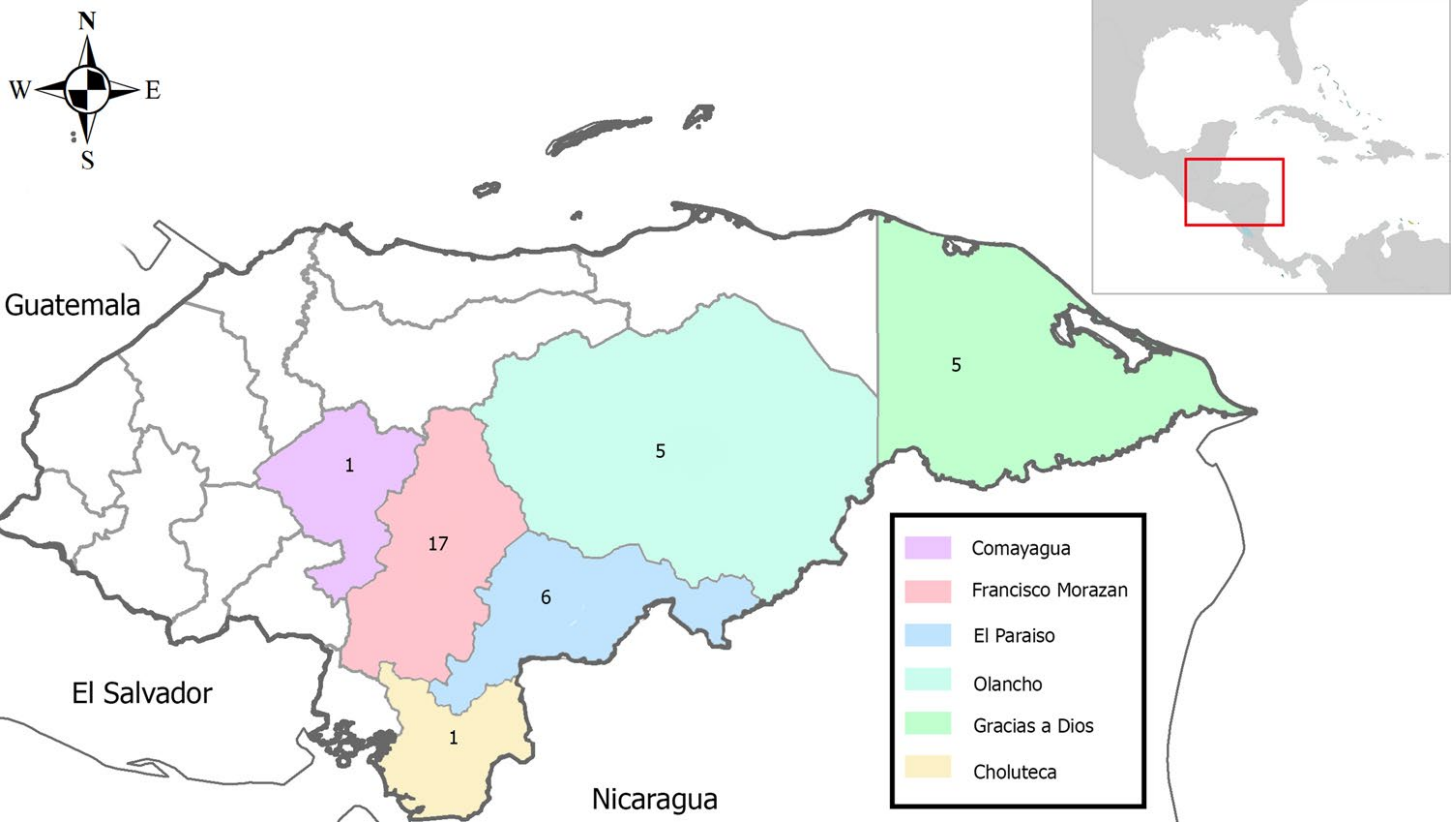
Distribution map of study cases. The colors on the map correspond to the Honduras States were patients came from and the numbers to the cases from each of the states. Most samples came from the department of Francisco Morazan where University Hospital is located. The map was created using open data obtained from GADM database of Global Administrative Areas, version 3.6. URL: http://www.gadm.org.

Blood spot samples were collected from 28 patients with *P. vivax* and 20 patients with *P. falciparum* monoinfection. From the 2005–2017 study period, nearly 50% of all *P. vivax* samples were collected between 2013 and 2014 whereas 50% of *P. falciparum* were collected between 2008 and 2010. Epidemiological data indicate that 21 *P. vivax* cases came from the regions of El Paraiso and Francisco Morazán whereas in *P. falciparum* 10 cases came from the departments of Olancho and Gracias a Dios (Fig. 1). In addition, epidemiological data from 3 *P. falciparum* cases suggests that they have been imported from Africa (Ghana, Congo and Kenya).

In *P. vivax,* barcode coverage for 23 out of the 28 samples were observed and data cleaning resulted in 7 out of 38 loci been removed due to missing values higher than 20%. In *P. falciparum*, barcode coverage for 16 out of the 20 samples were observed. However, 9 samples were removed for population structure and genetic analysis due to higher than 20% missing calls and also because three of them were suspected imported cases from Africa. Further data cleaning resulted in 16 out of 101 *P. falciparum* loci been removed due to missing values higher than 20%.

### Population diversity and complexity of infection

All *P. vivax* samples and all autochtonous *P. falciparum* samples were found to be monoclonal by both COIL and Real McCOIL methods. The median bootstrapping values of barcode intra population diversity (π) for *P. vivax* and *P. falciparum* were 0.25 and 0.07, respectively, which is indicative of a low parasite diversity.

### Drug resistance polymorphisms

In *P.* vivax, SNP genotyping showed that three samples isolated during different years presented the 976F mutation on *pvmdr1* that has been associated with CQ resistance^29^ whereas the remaining 20 were wildtype (976Y). Two of these subjects were recurrent cases previously diagnosed in the hospital. In addition, all samples presented the wild type haplotype 57F + 61S + 117T for *pvdhfr* and the wild type haplotype 383A + 553A for *pvdhps*. Mutations on these genes have been associated with pyrimethamine and sulfadoxine resistance, respectively^30,31^.

In *P. falciparum*, SNP genotyping showed that three samples (two imported and one local case) presented the CQ resistant haplotype SVMNT on *pfcrtr* (positions 72–76) (Table 2). The three samples have heterozygous SNPs on position 72 (C/S) and two of them were heterozygous at positions 74 and 75 (M/I and N/E) (Table 2). The first imported case was from a sample from 2008 from a Japanese subject who likely got the infection in Ghana (Africa) and cleared parasites at day 7 after mefloquine plus primaquine treatment.

**Table 2:**
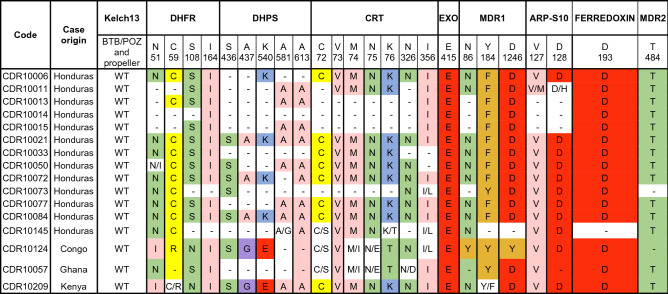
Multilocus drug resistance haplotypes.

The table shows SNPs on drug resistance genes on collected *P. falciparum* samples from our study. Colors on the amino acids indicate amino acid change according to the Zappo color scheme (Aliphatic/hydrophobic in pink, aromatic in orange, positive in blue, negative in red, hydrophilic in green, conformationally special in magenta and cysteine in yellow). Wild-type (WT), missing genotype (-) and heterozygous call (/).

The second imported case was from a sample collected in 2012 from a Honduran who likely got the infection in the Republic of Congo or Democratic Republic of Congo. This imported case also presented the *pfmdr1* 86Y mutation that further enhances the CQ resistant genotype^32–34^ (Table 2). This patient cleared parasites at day 13th post treatment with COARTEM. The local case with a CQ *pfcrt* resistant haplotype corresponded to a sample collected in 2013 from a subject from the region of Choluteca in Honduras whose therapeutic status was not assessed at that time.

Two samples were quintuple mutants carrying *pfdhfr* mutations at positions 51, 59 and 108 (IRN haplotype) and at positions 437 and 540 in *pfdhps* (GE haplotype) which have been strongly associated with sulfadoxine treatment failure^35^ (Table 2). One of these samples was from the Congo patient which held the *pfcrt* and *pfmdr1* resistant mutations as described. The other sample was from a child who was previously in Kenya and who cleared parasites on the fifth day after standard treatment.

All samples were wildtype for EXO which is associated with piperaquine resistance^36^ as well as for kelch13 artemisinin resistance associated mutations^37^. In addition, no evidence was found for parasite genetic background mutations (arps10, ferredoxin, pfmdr2 and pfcrt: 326, 356) that could allow the emergence of *k13* mutations^38^ (Table 2).

### Population structure

**Figure 2:**
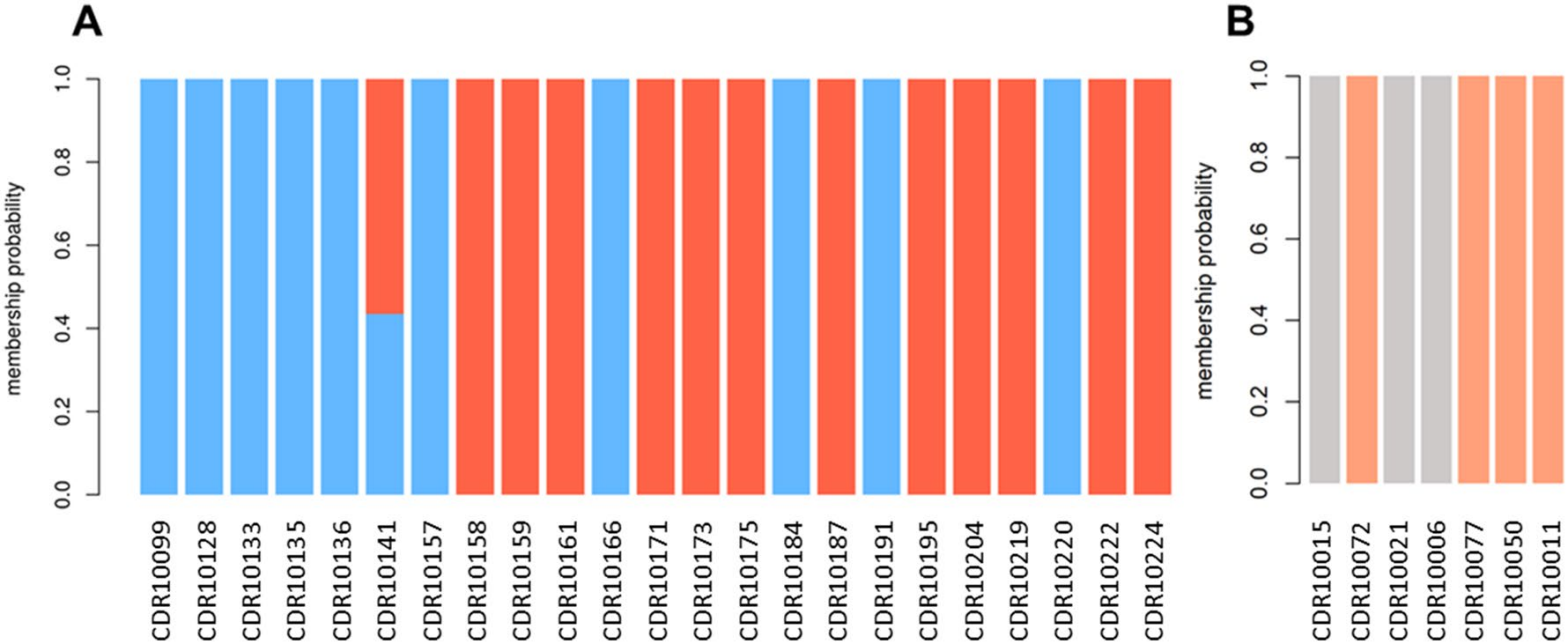
Clustering results of *P. vivax* and *P. falciparum*. The figure shows the population structure of 23 *P. vivax* samples (inset **A**) and 7 *P. falciparum* samples (inset **B**). The y-axis denotes the membership probability of each sample to a cluster whereas the color shows the two clusters that were identified for *P. vivax* and *P. falciparum*. The color scheme corresponds to the two clusters that k-means clustering assigned for each of the species.

Discriminant analysis of principal components did not found evidence of distribution according to geographical location nor time of collection for *P. vivax* (Supplementary figure 1). In the case of *P. falciparum*, the sample size was limited to see any patterns in the distribution. K-means clustering revealed the presence of two *P. vivax* and two *P. falciparum* parasite sub-populations with limited recombination between them (Fig. 2). In *P. falciparum*, only seven autochthonous samples were analyzed by this method due to the high rate of missing genotypes.

**Figure 3:**
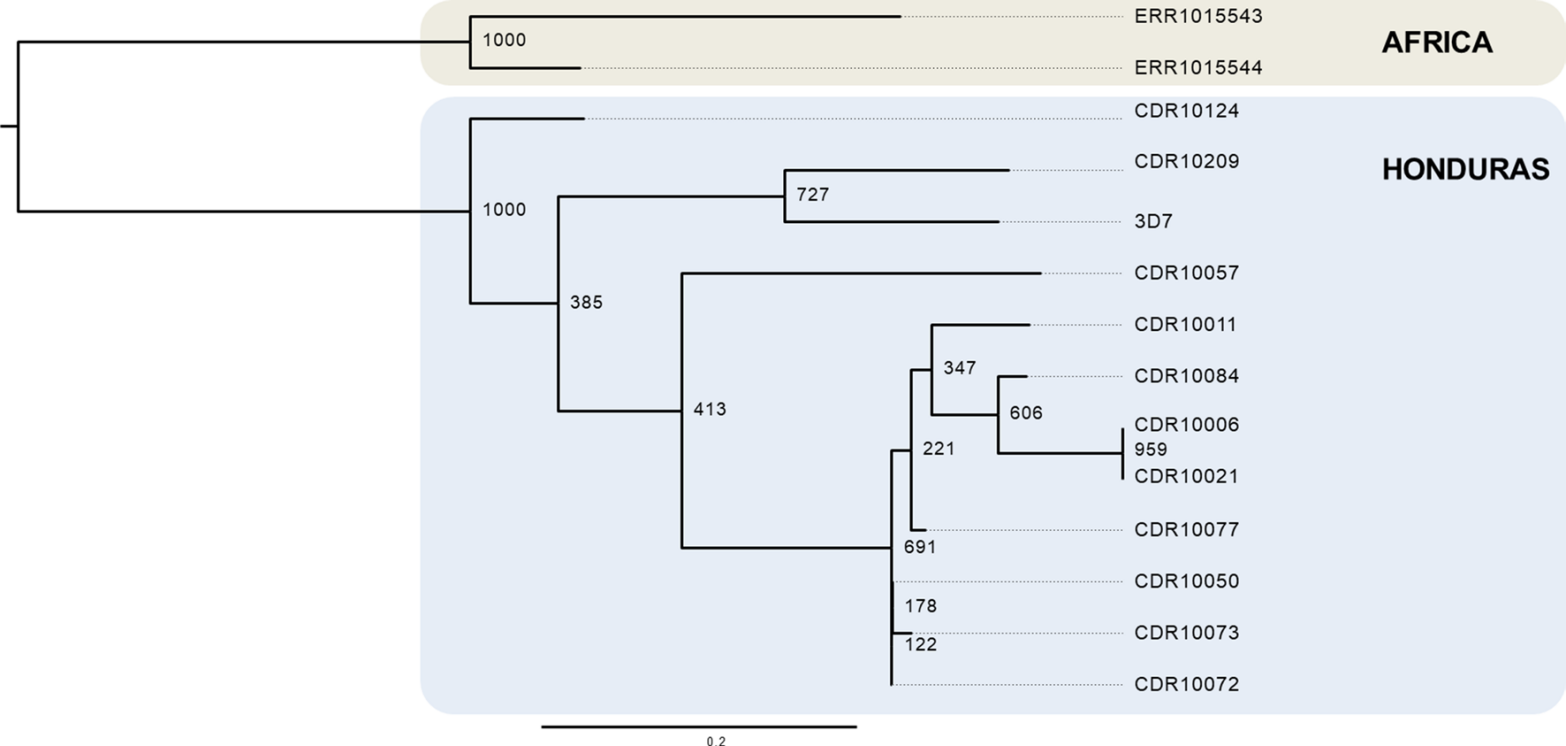
Maximum likelihood phylogenetic analysis. The figure shows that the three imported cases (CDR10124, CDR10209 and CDR10057) are closely related to the African and 3D7 strains rather than to Honduras autochthonous cases. Node numbers indicate bootstrap support.

Phylogenetic analyses showed that the three imported cases (CDR10124, CDR10209 and CDR10057) were genetically closer to African strains rather than to autochthonous cases which clustered in one clade (Fig. 3). The median-joining network showed that samples from Honduras, Colombia, Peru and Africa clustered according to their geographical location (Supplementary figure 2) as indicated by the single mutational-step separation. The three suspected imported samples from Honduras (CDR10124, CDR10209 and CDR10057) clustered separately from the rest of the Honduras samples and were closer to the African strains (Supplementary figure 2).

## Discussion

In malaria, polyclonal infections may arise from multiple infections from different mosquito bites with genetically unrelated parasites or infections with related parasites originated from a single meiosis event^39^. The genetic diversity that can result from these polyclonal infections could constitute a barrier for malaria elimination^39^. This is particularly important in a low transmission region like Honduras where these infections can lead to novel parasite variants that could potentially express more virulent phenotypes or contribute to the emergence and spread of drug resistance^40^.

In this regard, our results points towards a low parasite diversity with near 100% prevalence of monogenomic infections and a low barcode π (π = 0.25 and 0.07) for *P. vivax* and *P. falciparum*, respectively. This low level of genetic diversity might have been caused by a population bottleneck as a result of the highly effective intervention campaigns conducted in Honduras during the last two decades in the context of malaria elimination or by natural events such as hurricanes like Mitch in 1998 which swept most of Honduras. However, our sample sizes are too small to ensure an accurate estimate of genetic diversity.

In this regard, a previous study analyzed three markers for *P. vivax* (*pvama*, *pvcsp* and *pvmsp1*) and two markers for *P. falciparum* (*pfmsp1* and *pfsmp2*) on samples collected between 2010 and 2011 and showed a high genetic diversity for both species^41^. In *P. vivax*, that study reported 41, 23 and 23 genotypes for *pvama*, *pvcsp* and *pvmsp1*, respectively, whereas *P. falciparum* samples presented all three allelic families for *pfmsp1* (K1, MAD20, and RO33) and two allelic families for *pfmsp*2 (3D7 and FC27). However, their sample size was limited to accurately reflect the overall population diversity, especially for *P. falciparum*.

In contrast, another study based on neutral microsatellite markers in 110 *P. falciparum* isolates collected between 2009 and 2012 in Honduras and Nicaragua showed a low genetic diversity in both countries (0.35 and 0.38, respectively)^5^. Furthermore that study showed no evidence of *P. falciparum* population structure in Honduras with only one parasite cluster.

This apparent contradictory results might be explained by the different methods used by both studies and the distinct geographical regions that they covered. Nevertheless, the information provided by our and the previous studies highlights the need for more robust assessments including a larger sample size and the analysis of recent specimens given the reduction of malaria cases in Honduras during the last decade.

Our population structure analysis defined two putative clusters for *P. vivax* and *P. falciparum* which were not related to temporal nor spatial distribution of the isolates. In the case of the imported *P. falciparum* cases*,* our results showed that these strains were closely related with African circulating strains. This result match with the data collected from these cases which showed recent travel history to Africa and the presence of *pfdhfr* and *pfdhps* quintuple mutant genotypes associated with SP resistance.

Furthermore, our analyses of drug resistant genotypes showed that four *P. falciparum* samples presented either the CQ resistant haplotype SVMNT on *pfcrtr* or the *pfmdr1* 86Y mutation. Three of these samples were imported cases, one from an Honduran who worked in Africa, the other from a school-aged child^42^ and the third from a Japanese migrant with travel history to Africa. Due to their particular characteristics, these imported cases were treated with alternative drugs to chloroquine. The forth sample was a local case that harbored the SVMNT *pfcrt* CQ resistant haplotype whose therapeutic outcome was not assessed.

Conversely, 92% (12 out of 13) autochthonous *P. falciparum* cases were classified as wild type for all the tested drug resistance loci (Table 2). The evidence of wildtype genotypes in CQ drug resistant loci in our samples supports previous studies that showed that *P. falciparum* CQ resistance has not emerged nor been introduced in the country^5,9,43^. Furthermore, our findings are in alignment with clinical data from the country and indicate that CQ is still efficacious for the treatment of local *P. falciparum* cases in Honduras. However, the finding of a local case and three imported cases with drug resistant genotypes shows a potential risk for introduction of resistant strains and underscores the need for continuing surveillance.

It is important to mention that our study presented some weaknesses that need to be stated. The study sample size was rather small, focused on a specific site and limited time frame. Therefore, our results do not necessarily represent the pattern that could be seen in the country nor on the regions where the participants got infected.

Therefore, active and continuous monitoring of circulating drug resistance genotypes and phenotypes is critical to safeguard the progress that Honduras has achieved towards malaria elimination through early detection and monitor the risk of introduction of resistant strains in the country as well as for the Central America sub-region due to the wide interchange among the countries.

## Supplementary information

Supplementary Figures.

## Data Availability

The dataset generated during and/or analyzed during the current study are available from the corresponding author on reasonable request. Raw sequence data has been deposited at the European Nucleotide Archive (https://www.ebi.ac.uk/ena/browser/home) under ENA accession numbers ERS3475952 to ERS3475971 for *P. falciparum* and ERS3516634 to ERS3516661 for *P. vivax*.
