# Supplementary material for: Genomic surveillance of Plasmodium falciparum and Plasmodium vivax cases at the University Hospital in Tegucigalpa, Honduras
Source: Sci Rep. 2020 Dec 1;10:20975. doi: 10.1038/s41598-020-78103-w (PMC7708478; doi:10.1038/s41598-020-78103-w)
Supplement: Supplementary file 1 — Supplementary Figures. [file 41598_2020_78103_MOESM1_ESM.pdf]

Supplementary figures

Genomic surveillance of *Plasmodium falciparum* and *Plasmodium vivax* cases at the University Hospital in Tegucigalpa, Honduras

Hugo O. Valdivia, Fredy E. Villena, Stephen E. Lizewski, Jorge Garcia, Jackeline Alger, Danett K. Bishop

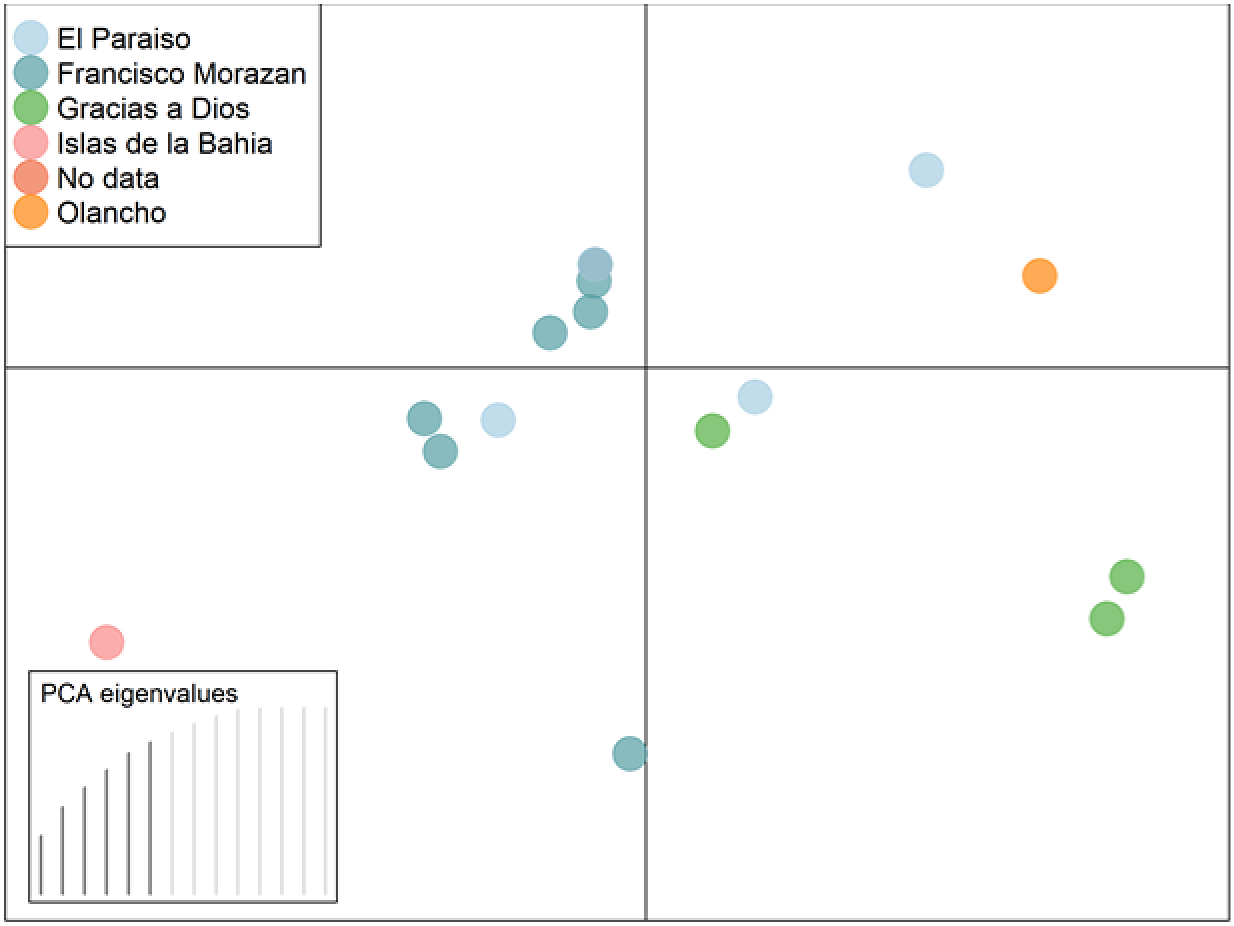

Supplementary figure 1. Discriminant analysis of principal components of Honduras *P. vivax* samples. The figure shows that there is no clustering of samples according to geographical location.

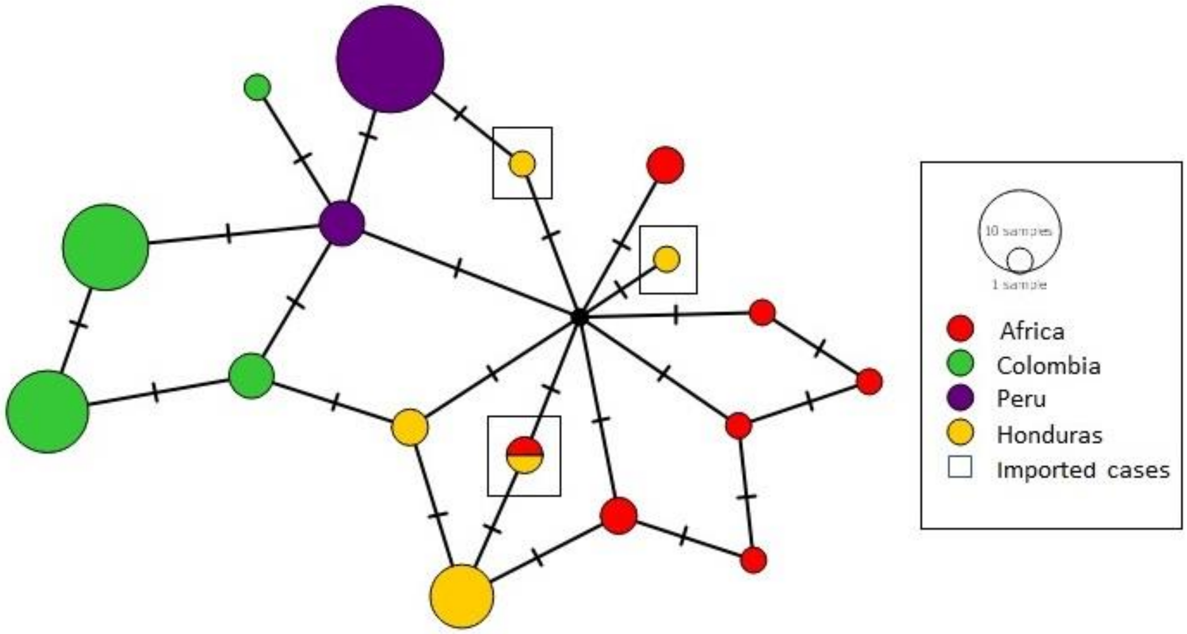

Supplementary figure 2: Median-joining network for *P. falciparum* samples from Honduras (yellow), Colombia (green), Peru (purple) and Africa (red). Sizes of the circles are proportional to the number of haplotype sequences. The number of straight lines connecting the haplotypes are proportional to the number mutational steps. The imported Honduras samples are marked in squares.
